# Development and characterization of SSR markers for *Sanguinaria canadensis* based on genome skimming

**DOI:** 10.1002/aps3.11289

**Published:** 2019-09-24

**Authors:** Renyu Liao, Yuxin Luo, Gulbar Yisilam, Ruisen Lu, Yuguo Wang, Pan Li

**Affiliations:** ^1^ Laboratory of Systematic and Evolutionary Botany and Biodiversity College of Life Sciences Zhejiang University Hangzhou Zhejiang 310058 People's Republic of China; ^2^ Ministry of Education Key Laboratory for Biodiversity Science and Ecological Engineering Institute of Biodiversity Science Fudan University Shanghai 200433 People's Republic of China

**Keywords:** eastern North America, *Eomecon*, microsatellites, Papaveraceae, *Sanguinaria canadensis*

## Abstract

**Premise:**

Polymorphic nuclear simple sequence repeat (nSSR) markers were developed for *Sanguinaria canadensis* (Papaveraceae), a spring ephemeral native to eastern North America.

**Methods and Results:**

Based on the genome skimming data of *S. canadensis*, a total of 240 nSSR primer pairs were designed for 80 loci from the assembled nuclear contigs. Of these primer pairs, 19 were selected for initial validation in four populations (80 individuals). All 19 loci produced heterologous amplification. The numbers of alleles per locus ranged from one to 21; the levels of observed and expected heterozygosity per locus ranged from 0.000 to 1.000 and from 0.000 to 0.847, respectively. Transferability of the loci was tested in the related species *Eomecon chionantha*.

**Conclusions:**

The developed nSSR markers revealed polymorphism in the four studied populations and may contribute to investigations of the genetic diversity of *S. canadensis* and *E. chionantha*.

*Sanguinaria canadensis* L. (bloodroot; Papaveraceae) is the only species of *Sanguinaria* L., a genus endemic to eastern North America. Bloodroot is a polymorphic species in regard to the morphology of its petals and stems, which vary greatly among individuals. Despite this polymorphism, most botanists consider the different phenotypes to represent variation within a single species; the variation is clearly continuous, exhibiting intermediate forms, and having no obvious correlation with either geography or habitat (Kiger, 1997). Bloodroot is a spring ephemeral herb commonly occurring in temperate deciduous forests, ranging from southern Ontario to eastern Texas in the west, and from northern Florida to New England in the east. Spring ephemerals exhibit a common strategy strongly associated with temperate deciduous forests that allows understory herbs to take advantage of the high levels of sunlight in spring reaching the forest floor prior to formation of a canopy by woody plants (Archibold, 1995). Understanding the population distribution and genetic structure of *S. canadensis* may not only shed light on the evolutionary history of the species, but also provide insights into the formation and evolution of the North American temperate deciduous forest biome.

Simple sequence repeats (SSRs) have been shown to be highly useful genetic markers for assessing genetic diversity and characterizing population genetic structure (Emanuelli et al., 2013; Lind and Gailing, 2013; Chen et al., 2017; Liu et al., 2018). However, there have been no SSR markers developed for *S. canadensis*. In this study, we developed 19 polymorphic nuclear SSR (nSSR) markers for *S. canadensis* using genome skimming and applied these markers to characterize the genetic diversity and population structure of four natural populations. Furthermore, we tested their cross‐amplification in the related species *Eomecon chionantha* Hance. Our results suggest that these nSSRs markers will be valuable for future studies on population genetics and phylogeography of *S. canadensis* across its entire geographic range.

## METHODS AND RESULTS

Two individuals of *S. canadensis* were selected for genome skimming analysis. Fresh leaves of these individuals (XGC1 and XGC2; Appendix 1) were sampled from the field and dried with silica gel. Total genomic DNA was extracted using Plant DNAzol Reagent (Thermo Fisher Scientific, Waltham, Massachusetts, USA) following the manufacturer's protocol. The high‐molecular‐weight DNA was sheared using a Covaris LE220 Focused‐ultrasonicator (Covaris, Woburn, Massachusetts, USA), then the library was prepared using a NEBNext DNA Library Prep Master Mix Set for Illumina (New England Biolabs, Ipswich, Massachusetts, USA), followed by a size selection of 300–350 bp using the Agencourt AMPure XP system (Beckman Coulter, Shanghai, China). Finally, sequencing was conducted using the Illumina HiSeq 2500 (Illumina, San Diego, California, USA) at the Beijing Genomics Institute (Shenzhen, China), with 150‐bp paired‐end sequencing. The raw reads were filtered and then assembled de novo into contigs using the CLC Genome Workbench version 4.0.6 (QIAGEN, Hilden, Germany), following Liu et al. (2017). The raw reads were deposited in the National Center for Biotechnology Information (NCBI) Sequence Read Archive (BioProject ID: PRJNA512069 for XGC1, PRJNA512066 for XGC2).

Contigs mapped to the plastome of *Coreanomecon hylomeconoides* Nakai (NC_031446) and the mitochondria of *Nelumbo nucifera* Gaertn. (NC_030753) were removed, so that only nuclear contigs remained. BLAST 2.2.26 (Altschul et al., 1990) was used for remapping. The remaining nuclear contigs were analyzed using CandiSSR (Xia et al., 2016) to identify candidate polymorphic nSSRs by comparing the contigs between the two *S. canadensis* individuals. For CandiSSR analysis, default parameters were used except that flanking sequence length = 50. For each SSR locus, primers were automatically designed in the pipeline via the Primer3 package (Koressaar and Remm, 2007), which generated three primer pairs for each of the 80 loci. The 240 SSR primer pairs (Table 1, Appendix S1) were then evaluated by OLIGO version 6.67 (Molecular Biology Insights, Cascade, Colorado, USA); primers that had ΔG values <4.5 kcal/mol and no annealing troubles were selected.

**Table 1:** Characteristics of 19 microsatellite loci developed for *Sanguinaria canadensis*.

Locus	Primer sequences (5′–3′)	Repeat motif	Allele size range (bp)	*T*_a_ (°C)	*A*	GenBank accession no.
SSR04	F: TGCATATTCACAACCGTTGAGC	(AATT)_6_	142–183	59.7	8	MK369726
	R: CGCTGGAACCTGCAAATTGA					
SSR05	F: TGTGTAAGAGTGCTAACCCTCA	(AC)_6_	134–146	55.8	4	MK369728
	R: GGTTCGGCCAATTCGGTCTA					
SSR11	F: TCCCTTCAAGATCACAGCGT	(AG)_6_	118–134	55.8	9	MK369735
	R: CTTAGCTTCGCCGGTACACA					
SSR12	F: TCATTCGTGGCTGCCTTACA	(AG)_7_	135–137	55.8	2	MK369732
	R: ACCATCGATTACAACCATCCCT					
SSR18	F: CCTCCTCCCTGAACATAACCC	(AG)_7_	187–197	57.1	3	MK369724
	R: GATGCTTCCATGGGGAGGAC					
SSR41	F: AGCAGGTGCAGCAATCTTCT	(CAG)_6_	157–163	59.7	3	MK369727
	R: GCCATCACGAGTCCAACGTA					
SSR42	F: ATCGTGCGATCCTTCCCATC	(CAG)_6_	154–163	55.8	3	MK369719
	R: TGCTAGTTCTGTCGGCACTG					
SSR44	F: CTTCTTTCACCCTCGAACGC	(CT)_20_	181–239	55.8	21	MK369725
	R: ATTTCCCACGGCCACTTCTT					
SSR51	F: ACCCTCACTTCTATGTTCACTCC	(CTC)_7_	155–170	58.4	5	MK369729
	R: CAAGGCAAGAAAGGCAGCAG					
SSR56	F: ACAATCGACCTGCAACGAG	(GA)_13_	130–162	58.4	13	MK369731
	R: CGGTTCAGATCTGTGAGTCGA					
SSR59	F: GTTCTTGAGGTGAAGTTATTCG	(GA)_6_	114–134	55.8	3	MK369723
	R: AGGGGAGACTGTCGGATTTG					
SSR64	F: AACCCAGGAGAGCAAACCAT	(GAT)_6_	165–174	61.9	4	MK369720
	R: CCACCCTTCTCACCATCGTC					
SSR67	F: CACGAGTTGTGTCCGGAATT	(GTG)_5_	131–143	58.4	4	MK369736
	R: TCGTCGCTCTGCTTCATCTT					
SSR68	F: TGTTGGCTGGCATTCTTGTTC	(GTTT)_5_	189	60.9	1	MK369733
	R: GGCATGCTGAAAAGCTCGTG					
SSR80	F: TGAAAGAATCAGACATGGCT	(TC)_7_	123–131	57.1	5	MK369730
	R: GCTGGTGTGGTAATCCTGTC					
SSR90	F: CTACAGACGTTCGCGAGGTA	(TCT)_5_	109–121	53.8	5	MK369718
	R: ATCCGCAACTACGCACGTAA					
SSR92	F: GCAACCACCAACAGATGACG	(TCT)_9_	166–184	59.7	7	MK369722
	R: AGCTTCTTGTTTCCAGGCAAG					
SSR93	F: TTCAACAGCAGGCGTCTTCT	(TCT)_9_	110–128	54.7	5	MK369721
	R: TGCCGAAGCAGCATCCATTA					
SSR94	F: ACTTGATCCTTCTGCTCCGT	(TGA)_8_	143–173	62.6	7	MK369734
	R: CCTTCTTGTGGTGCTAACTGC					

Note

*A* = total number of alleles per locus detected in the four populations; *T*_a_ = optimized annealing temperature.

In total, 19 primer pairs were selected to test in 80 individuals from four natural populations (20 individuals each) of *S. canadensis* (Appendix 1; Tables 1, 2). DNA was extracted from the leaf tissues as described above. Two rounds of PCR amplifications were performed using a Tsingke PCR kit (Tsingke Biotech Company, Beijing, China). For the first round, a mixture of 15 μL containing approximately 5 ng DNA, 7.5 μL 2 × PCR Master Mix, 5 pM forward primer (synthesized with an 18‐bp M13 tail [5′‐TGTAAAACGACGGCCAGT‐3′] at the 5′ end), and 5 pM of reverse primer. The PCR thermal profile involved an initial denaturation at 95°C for 5 min; followed by 35 cycles of denaturation at 95°C for 30 s, annealing at optimal temperature (Table 1) for 30 s, and extension at 72°C for 30 s; and a final extension of 72°C for 10 min. For the second round, a final volume of 30 μL contained approximately 3 μL of the first round's product, 15 μL of 2× PCR Master Mix, 100 μM of fluorescent‐labeled (FAM, ROX, HEX, or TAMRA) universal M13 primer, and 10 pM of reverse primer. The PCR reactions were performed with an initial denaturation at 94°C for 2 min; followed by 38 cycles of 94°C for 60 s, 56°C for 45 s, 72°C for 1 min; and a final 10‐min extension step at 72°C. The first round of PCR added an adapter to the fragments, which were then fluorescent‐labeled in the second round of PCR. The final amplification products of four different loci (with four different fluorescent labels) for each plant sample were pooled and genotyped in an ABI 3730xl DNA Analyzer (Applied Biosystems, Foster City, California, USA) with GeneScan 500 LIZ Size Standard (Applied Biosystems) as the internal reference.

**Table 2:** The genetic parameters (per locus) of 19 microsatellite loci in four populations of *Sanguinaria canadensis*.^a^

Locus	WI (*n* = 20)				MN (*n* = 20)				NC (*n* = 20)				VA (*n* = 20)			
	*A*	*H*_o_	*H*_e_	PIC	*A*	*H*_o_	*H*_e_	PIC	*A*	*H*_o_	*H*_e_	PIC	*A*	*H*_o_	*H*_e_	PIC
SSR04	4	0.400	0.353	0.323	2	0.600	0.431	0.332	5^c^	0.750	0.619	0.529	6	0.700	0.664	0.602
SSR05	4	0.600	0.483	0.433	3	0.700	0.518	0.442	3	0.850	0.555	0.439	4	0.600	0.545	0.491
SSR11	7	0.950	0.781	0.725	8	0.900	0.812	0.765	4	0.789	0.575	0.488	5	0.842	0.747	0.687
SSR12	1	0.000	0.000	0.000	1	0.000	0.000	0.000	1	0.000	0.000	0.000	2	0.600	0.513	0.375
SSR18	2	0.450	0.358	0.288	2	0.150	0.142	0.129	3	0.250^b^	0.501^b^	0.437	3^c^	0.316^b^	0.592^b^	0.513
SSR41	2	0.250	0.224	0.195	2	0.050	0.050	0.048	3	0.350	0.304	0.265	3	0.750	0.514	0.416
SSR42	2	0.350	0.296	0.247	2	0.150	0.142	0.129	1	0.000	0.000	0.000	2	0.400	0.385	0.305
SSR44	9	0.700	0.694	0.630	9	0.650	0.631	0.597	12	1.000	0.847	0.808	9	0.947	0.846	0.802
SSR51	4	0.900	0.594	0.497	4	0.350	0.350	0.317	4	1.000	0.692	0.619	5	0.900	0.763	0.702
SSR56	6	1.000	0.723	0.670	9	1.000	0.799	0.752	4	1.000	0.581	0.473	9	1.000	0.842	0.800
SSR59	3	0.350	0.309	0.276	2	0.050	0.050	0.048	2	0.050	0.050	0.048	3	0.150	0.145	0.136
SSR64	2	0.300	0.262	0.222	1	0.000	0.000	0.000	3	0.150	0.232	0.214	2^c^	0.350	0.409	0.319
SSR67	2	0.800	0.492	0.365	2	0.850	0.501	0.369	4	0.900	0.568	0.466	2	0.950	0.512	0.374
SSR68	1	0.000	0.000	0.000	1	0.000	0.000	0.000	1	0.000	0.000	0.000	1	0.000	0.000	0.000
SSR80	2^c^	0.050^b^	0.224^b^	0.195	1	0.000	0.000	0.000	5	0.600	0.629	0.575	4	0.600	0.506	0.447
SSR90	3	0.500	0.559	0.441	3^c^	0.150^b^	0.337^b^	0.289	2	0.050	0.050	0.048	3	0.550	0.555	0.439
SSR92	5	0.950	0.683	0.614	3	0.950	0.560	0.442	5	1.000	0.678	0.598	5	0.750	0.618	0.557
SSR93	2	1.000	0.513	0.375	2	1.000	0.513	0.375	2	1.000	0.513	0.375	5	1.000	0.733	0.663
SSR94	3	0.300	0.272	0.247	2	0.400	0.431	0.332	6	1.000	0.708	0.657	5	0.900	0.653	0.597
Mean	3.44	0.518	0.412	0.364	3.11	0.418	0.330	0.282	3.61	0.565	0.426	0.367	4.35	0.650	0.555	0.485

Note

*A* = number of alleles per locus detected in each population; *H*_e_ = expected heterozygosity; *H*_o_ = observed heterozygosity; *n* = number of individuals sampled; PIC = polymorphism information content.

a Voucher and locality information are provided in Appendix 1.

b Significant deviations (*P* < 0.05) from Hardy–Weinberg equilibrium detected in the population.

c Estimated null allele frequency *r* > 0.05.

Allele identification was performed using GeneMarker version 2.2.0 (SoftGenetics, State College, Pennsylvania, USA) with default parameters. The presence of null alleles and their bias on genetic diversity were evaluated based on the expectation maximization method implemented in FreeNA (Chapuis and Estoup, 2006). FSTAT version 2.9.3 (Goudet, 1995) was used to test for Hardy–Weinberg equilibrium, and GENEPOP version 4.0.7 (Rousset, 2008) was used to test for linkage disequilibrium. The number of alleles, levels of observed and expected heterozygosity, and polymorphism information content were calculated using CERVUS version 3.0.3 (Kalinowski et al., 2007).

In total, we detected 112 alleles among 19 loci of 80 individuals. The number of alleles at each locus ranged from one to 21, and PIC values ranged from 0.000 to 0.808 (Table 2), suggesting moderate to high levels of polymorphism (Botstein et al., 1980). Levels of observed and expected heterozygosity for each locus varied from 0.000 to 1.000 and 0.000 to 0.847, respectively (Table 2). These data suggest the presence of abundant genetic diversity in the species. Five loci (SSR4, SSR18, SSR64, SSR80, and SSR90) were shown to have null alleles (Table 2; Chapuis and Estoup, 2006). Loci SSR18, SSR80, and SSR90 revealed significant deviation from Hardy–Weinberg equilibrium in populations NC and VA, population WI, and population MN (*P* > 0.05; Table 2), respectively. Significant linkage disequilibrium was only detected between loci SSR18 and SSR92. These data may indicate non‐random mating, and possibly inbreeding, of the plants in these populations.

To verify their potential for heterologous amplification, all 19 SSRs were tested in one population (20 individuals) of *E. chionantha* (Appendix 1), the sister species of *S. canadensis* (Wang et al., 2009). The procedures of PCR amplification and genotyping were the same as described above. All 19 loci amplified in *E. chionantha*, with polymorphism detected in all loci except SSR68 (Table 3), suggesting their potential utility in future studies.

**Table 3:** Fragment sizes detected in cross‐amplification tests of 19 microsatellite markers developed for *Sanguinaria canadensis* in one population (*n* = 20) of *Eomecon chionantha*.^a^

Locus	Allele size range (bp)
SSR4	147–171
SSR5	134–146
SSR11	118–120
SSR12	125–151
SSR18	181–197
SSR41	157–163
SSR42	151–157
SSR44	183–199
SSR51	161–167
SSR56	144–150
SSR59	110–134
SSR64	156–162
SSR67	128–137
SSR68	181
SSR80	117–125
SSR90	109–130
SSR92	169–184
SSR93	110–128
SSR94	143–170

a Voucher and locality information are provided in Appendix 1.

## CONCLUSIONS

In this study, 19 highly polymorphic nSSR markers were successfully developed for *S. canadensis*. These markers can be applied to investigate population genetics and phylogeography of *S. canadensis* and its close relatives.

## Supporting information


**APPENDIX S1.** The remaining 221 primers developed through the pipeline for *Sanguinaria canadensis*.

## Data Availability

Raw sequencing reads were deposited in the National Center for Biotechnology Information (NCBI) Sequence Read Archive (BioProject ID: PRJNA512069 for XGC1, PRJNA512066 for XGC2). Sequence information for the developed primers has been deposited in NCBI's GenBank, and accession numbers are provided in Table 1.

## Voucher information for species used in this study.

**Table:**

Species	Population code	Voucher no.^a^	Locality	Geographic coordinates	Altitude (m)	*n*
*Sanguinaria canadensis* L.	XGC1	*Pan Li, LP162238*	Winslow, Arkansas, USA	35.78180°N, 94.21827°W	350	
	XGC2	*Pan Li, LP162495*	Marianna, Florida, USA	30.79882°N, 85.22835°W	21	
	WI	*Pan Li, LP185609*	Lyndon Station, Wisconsin, USA	43.67250°N, 90.03027°W	380	20
	MN	*Pan Li, LP185619*	Rochester, Minnesota, USA	44.03513°N, 92.43248°W	320	20
	NC	*Pan Li, LP185735*	Micaville, North Carolina, USA	35.81235°N, 82.14401°W	1136	20
	VA	*Pan Li, LP185767*	McLean, Virginia, USA	38.96440°N, 77.15205°W	68	20
*Eomecon chionantha* Hance	CQ	*Pan Li, LP185255*	Nanchuan, Chongqing, China	29.11748°N, 107.33582°E	1289	20

Note

*n* = number of individuals per population.

a Vouchers were deposited in the Herbarium of Zhejiang University (HZU), Hangzhou, Zhejiang, China.
