# Supplementary material for: Development and characterization of SSR markers for Sanguinaria canadensis based on genome skimming
Source: Appl Plant Sci. 2019 Sep 24;7(9):e11289. doi: 10.1002/aps3.11289 (PMC6764431; doi:10.1002/aps3.11289)
Supplement: Supplementary file 1 — APPENDIX S1. The remaining 221 primers developed through the pipeline for Sanguinaria canadensis. [file APS3-7-e11289-s001.docx]

**APPENDIX S1.** The remaining 221 primers developed through the pipeline for *Sanguinaria canadensis*.

| **Locus** | **Forward primer (5′–3′)** | **Reverse primer (5′–3′)** |
| --- | --- | --- |
| SSR01 | AAGCACCCAGTTCTGACACC | GACTGGGTACTCGTTAGTGGT |
|  | AAGCACCCAGTTCTGACACC | AGACTGGGTACTCGTTAGTGGT |
|  | AAGCACCCAGTTCTGACACC | ACTGGGTACTCGTTAGTGGT |
| SSR02 | CGGATCAGCAGCATATCGCA | AGTTGCTTTGGCTTTTGGTTGT |
|  | CGGATCAGCAGCATATCGCA | GTTGCTTTGGCTTTTGGTTGT |
|  | AGTGCATTAGTCTGTGCGTGT | AGTTGCTTTGGCTTTTGGTTGT |
| SSR03 | ACGGCTGAAGAAGGAAGGAG | GTGGAGTTGCAGGTCCTTCA |
|  | ACGGCTGAAGAAGGAAGGAG | GCCCAAACAACAGCAACAGT |
|  | ACGGCTGAAGAAGGAAGGAG | GCAGGTCCTTCAATGGTTGC |
| SSR04 | TGCATATTCACAACCGTTGAGC | TCGCTGGAACCTGCAAATTG |
|  | TGCATATTCACAACCGTTGAGC | CATTCGCTGGAACCTGCAAA |
| SSR05 | TTGTGTAAGAGTGCTAACCCTCA | GGTTCGGCCAATTCGGTCTA |
|  | TGTGTAAGAGTGCTAACCCTCA | TTCGGCCAATTCGGTCTACA |
| SSR06 | CCAACAAGTGGGGTTTCAATCA | GGGCTTTCCCGCATACCATA |
|  | CCAACAAGTGGGGTTTCAATCAA | GGGCTTTCCCGCATACCATA |
|  | CCAACAAGTGGGGTTTCAATC | GGGCTTTCCCGCATACCATA |
| SSR07 | ACTCCCTCTCGCTCTCCATT | TTGAATCAGGTAGGCGGCTT |
|  | TCCCTCTCGCTCTCCATTGA | TTGAATCAGGTAGGCGGCTT |
|  | CTACTCCCTCTCGCTCTCCA | TTGAATCAGGTAGGCGGCTT |
| SSR09 | TAGCAGCAGGTGAGCAGTTC | TGCACGACCAGGAAGAGAAG |
|  | TAGCAGCAGGTGAGCAGTTC | CTGCACGACCAGGAAGAGAA |
|  | CAGCAGGTGAGCAGTTCTCT | TGCACGACCAGGAAGAGAAG |
| SSR10 | GAACTGGGAGTGATGTGCGA | CAACAAAGGCTGCATGGCA |
|  | GAACTGGGAGTGATGTGCGA | AGCAACAAAGGCTGCATGG |
|  | CTGGGAGTGATGTGCGAGAT | CAACAAAGGCTGCATGGCA |
| SSR11 | TCCCTTCAAGATCACAGCGT | AGCTTCGCCGGTACACATAC |
|  | TCCCTTCAAGATCACAGCGT | CTCCTTAGCTTCGCCGGTAC |
| SSR12 | TCATTCGTGGCTGCCTTACA | AACCATCGATTACAACCATCCCT |
|  | TCATTCGTGGCTGCCTTACA | ACCATCGATTACAACCATCCC |
| SSR14 | CCACCAACGGCACATTTCAC | GCGCGTTGAGAAACCAGAAG |
|  | CACCAACGGCACATTTCACA | GCGCGTTGAGAAACCAGAAG |
|  | AACGGCACATTTCACAAGCT | GCGCGTTGAGAAACCAGAAG |
| SSR16 | CCCCTAGGAGCAGTAGCAGA | GGAGCTCTCGTGTGATCCAG |
|  | CCCCTAGGAGCAGTAGCAGA | ACAGAGGAGCTCTCGTGTGA |
|  | CCCCTAGGAGCAGTAGCAGA | AGGAGCTCTCGTGTGATCCA |
| SSR17 | AGTGCACTGGACTTGTGTGA | ACACTTACACACCGACCATGA |
|  | AGTGCACTGGACTTGTGTGA | TAGACCAAAATGGCCGAACC |
|  | AGTGCACTGGACTTGTGTGA | TGGCCGAACCACGTAAAATAC |
| SSR18 | CTCCCTGAACATAACCCACCA | GATGCTTCCATGGGGAGGAC |
|  | CCTCCTCCCTGAACATAACCC | AGATGCTTCCATGGGGAGGA |
| SSR19 | CAGAGAACCTCAGCCACCAC | CTGGGGCCTGAGTGTAAAGG |
|  | CAGAGAACCTCAGCCACCAC | AGTAGAACTGGGGCCTGAGT |
|  | CAGAGAACCTCAGCCACCAC | AGAACTGGGGCCTGAGTGTA |
| SSR20 | AAAAGACCAGGGTGCATA | CGCAGATGCCCTGAGAATTA |
|  | AAAAGACCAGGGTGCATA | TGCTCTAAGAGGACAGGACA |
|  | AAAAGACCAGGGTGCATA | CGCAGATGCCCTGAGAATT |
| SSR23 | TTCCAATCGATCGGCCAACA | CTGATGCTGCTGCCATGTTT |
|  | GATCGGCCAACAGAGTAGCA | CTGATGCTGCTGCCATGTTT |
|  | ATCGGCCAACAGAGTAGCAG | CTGATGCTGCTGCCATGTTT |
| SSR24 | AGTTGGGCCACAAAGTCGTT | CGACTGCTGAAGTTGCAACA |
|  | AAGTTGGGCCACAAAGTCGT | CGACTGCTGAAGTTGCAACA |
|  | GGAGGATAAGTTGGGCCACA | CGACTGCTGAAGTTGCAACA |
| SSR26 | TTATCCATTGGCCGCAGCTC | GATGGTCCCCAGGAAAGTGT |
|  | TTATCCATTGGCCGCAGCTC | CCAAGATGGTCCCCAGGAAA |
|  | CGCAGCTCTCTCCTATGATGG | GATGGTCCCCAGGAAAGTGT |
| SSR32 | CGACGCCTTATGCCAACATG | TCGAAGCAACAACCAGATCA |
|  | TCGACGCCTTATGCCAACAT | TCGAAGCAACAACCAGATCA |
|  | AAGACTCGACGCCTTATGCC | TCGAAGCAACAACCAGATCA |
| SSR33 | GCTCAGTAGTAAAGGTCTGGCA | TGGTCACCTTGGTGATGCAG |
|  | GCTCAGTAGTAAAGGTCTGGCA | CTGGTCACCTTGGTGATGCA |
|  | GCTCAGTAGTAAAGGTCTGGCA | GGTCACCTTGGTGATGCAGT |
| SSR34 | TCTGCCTGGTAAGGGTTAGGA | TGAGACTGATCAAAATGAGGCTGA |
|  | TCTGCCTGGTAAGGGTTAGGA | GGCTGATATTTTCTACTCCATGGC |
|  | TCTGCCTGGTAAGGGTTAGGA | GAGACTGATCAAAATGAGGCTGA |
| SSR35 | CAAGTGGGGCATGGAGCTTA | AGCGTTCAATGAAAGTGGAGT |
|  | CAAGTGGGGCATGGAGCTTA | AGCGTTCAATGAAAGTGGAGTTG |
|  | CAAGTGGGGCATGGAGCTTA | AGCGTTCAATGAAAGTGGAGTT |
| SSR36 | TTGGTGCACATGGTAGGGTT | CCGATGCTCTGGGTTCTTCC |
|  | TTTGGTGCACATGGTAGGGT | CCGATGCTCTGGGTTCTTCC |
|  | TCGTGAAGTTGCTGCCACAA | CCGATGCTCTGGGTTCTTCC |
| SSR37 | GGCACTGTCAAGCTCTGGAA | GCCATGCATGCATCAAGAGG |
|  | GGCACTGTCAAGCTCTGGAA | GGCCATGCATGCATCAAGAG |
|  | GGCACTGTCAAGCTCTGGAA | GAGGCCATGCATGCATCAAG |
| SSR38 | GAATTCATGTATTGGCTAGGAC | TTGGGCCTGGATCCGAATAG |
|  | GAATTCATGTATTGGCTAGGAC | CTTGGGCCTGGATCCGAATA |
|  | GAATTCATGTATTGGCTAGGAC | ATCCGAATAGGGTCCATGGG |
| SSR39 | TGCTCATTATATGCAGATCAGCCT | TGGTTCTTTTCTCTGTTGTTCGG |
|  | AGGTTCAGAAGAAATTGCTTGCTC | TGGTTCTTTTCTCTGTTGTTCGG |
|  | GGTTCAGAAGAAATTGCTTGCTC | TGGTTCTTTTCTCTGTTGTTCGG |
| SSR40 | TGGAGAGGCTAGTACTTGGGA | AGAGGGTCTTGATGCAATTTCA |
|  | GTGGAGAGGCTAGTACTTGGG | AGAGGGTCTTGATGCAATTTCA |
|  | TGGAGAGGCTAGTACTTGGGA | GAGGGTCTTGATGCAATTTCA |
| SSR41 | CAGCAGGTGCAGCAATCTTC | GCCATCACGAGTCCAACGTA |
|  | ATGCAAGCAGGTGGTGGATT | GCCATCACGAGTCCAACGTA |
| SSR42 | ATCGTGCGATCCTTCCCATC | TAGTTCTGTCGGCACTGCTG |
|  | ACCACTGGCCCCACTACTTA | TAGTTCTGTCGGCACTGCTG |
| SSR43 | ATCTCCATTTCCGGCACAGT | ACGAACCCAAAATGGAGGAAGA |
|  | CATCTCCATTTCCGGCACAG | ACGAACCCAAAATGGAGGAAGA |
|  | ATCTCCATTTCCGGCACAGT | ACGAACCCAAAATGGAGGAAG |
| SSR44 | CTTCTTTCACCCTCGAACGC | CGGAGAAGCTCTGGTTCCTG |
|  | CTTCTTTCACCCTCGAACGC | AATTTCCCACGGCCACTTCT |
| SSR45 | CGCATTCTATGGGACGAGGT | TCGATCACTCAGGTCAGATTGT |
|  | CGCATTCTATGGGACGAGGT | TGAATCGATCACTCAGGTCAGA |
|  | CGCATTCTATGGGACGAGGT | CGATCACTCAGGTCAGATTGT |
| SSR46 | GGATTTGCCAGTGCTCGTTC | GCTGAGGCTGTCGTCTTTTG |
|  | TGGATTTGCCAGTGCTCGTT | GCTGAGGCTGTCGTCTTTTG |
|  | GGATTTGCCAGTGCTCGTTC | TCCGAAAGAGTGCAAGAGGT |
| SSR47 | GGTGTGAAATCGAGTGGAGGT | GTCCACTCCCCACAGATTGG |
|  | GGTGTGAAATCGAGTGGAGGT | TCCACACCGGCTTCTTTACC |
|  | GGTGTGAAATCGAGTGGAGGT | CCACACCGGCTTCTTTACCT |
| SSR48 | GGTCGGCGGATATCCTTAGC | CACCATCAGTAGGCGCAAGA |
|  | AGGCTAATAGGTCGGCGGAT | CACCATCAGTAGGCGCAAGA |
|  | GTCGGCGGATATCCTTAGCA | CACCATCAGTAGGCGCAAGA |
| SSR49 | ACCGATCAAGTTCCCCTGAA | GAATCTAGTACTCCAAACACAC |
|  | ACCGATCAAGTTCCCCTGAAT | GAATCTAGTACTCCAAACACAC |
|  | CTTAACCCCATGCCCAGGA | GAATCTAGTACTCCAAACACAC |
| SSR50 | CTGCCTGCTGTACCCTGTTA | TCAAGTCGTGCCCACTTCTC |
|  | CTGCCTGCTGTACCCTGTTA | CAAGTCGTGCCCACTTCTCT |
|  | CTGCCTGCTGTACCCTGTTA | CTCAAGTCGTGCCCACTTCT |
| SSR51 | CCCTCACTTCTATGTTCACTCCA | CAAGGCAAGAAAGGCAGCAG |
|  | CCCTCACTTCTATGTTCACTCCA | CAGCGAACCACTGGAGAAGA |
| SSR53 | AGAGAGGAGGAAGGGAAGGC | ACAGCAGCAGTTCTCCTTCG |
|  | AGAGAGGAGGAAGGGAAGGC | GGTTTCACGGCAGCAGTAGA |
|  | AGAGAGGAGGAAGGGAAGGC | TGGTTTCACGGCAGCAGTAG |
| SSR54 | TCTCGAGATCAGATCCGAGGT | AGATCCGATCTGACGTGGAGA |
|  | TCTCGAGATCAGATCCGAGGT | GATCCGATCTGACGTGGAGA |
|  | TCTCGAGATCAGATCCGAGGT | AGATCCGATCTGACGTGGAG |
| SSR55 | GAGGTGCCCTAGAAAGAACCA | CGTATGTCTATGGGCAGGAGA |
|  | AGGTGCCCTAGAAAGAACCA | CGTATGTCTATGGGCAGGAGA |
|  | AGAGGTGCCCTAGAAAGAACC | CGTATGTCTATGGGCAGGAGA |
| SSR56 | ACAATCGACCTGCAACGAGT | ACGGTTCAGATCTGTGAGTCG |
|  | AGACAATCGACCTGCAACGA | ACGGTTCAGATCTGTGAGTCG |
| SSR57 | TGGTCCCCACTCTTCTCTGA | ATTCAGCTGTGGGGTCCATG |
|  | GGTCCCCACTCTTCTCTGAC | ATTCAGCTGTGGGGTCCATG |
|  | TGGTCCCCACTCTTCTCTGA | TGTGGGGTCCATGTTGTTGG |
| SSR58 | GGAGATCAAACAGATTCAACGAGA | CCACCGGTACTGCTCATAGG |
|  | AGGAGATCAAACAGATTCAACGAG | CCACCGGTACTGCTCATAGG |
|  | GGAGATCAAACAGATTCAACGAG | CCACCGGTACTGCTCATAGG |
| SSR59 | GTTCTTGAGGTGAAGTTATTCG | GGGGAGACTGTCGGATTTGA |
|  | GTTCTTGAGGTGAAGTTATTCG | GGGGAGACTGTCGGATTTGAG |
| SSR60 | CAGGAACAGAGGAAGCTAAGGG | ACACATTGGAGGACGATACCG |
|  | GGAACAGAGGAAGCTAAGGGA | ACACATTGGAGGACGATACCG |
|  | AGGAACAGAGGAAGCTAAGGG | ACACATTGGAGGACGATACCG |
| SSR62 | GCTCAGGTACGACAAAACCC | TCCTGATGATCGAGCTCAGC |
|  | GCTCAGGTACGACAAAACCC | CCTGATGATCGAGCTCAGCT |
|  | GCTCAGGTACGACAAAACCC | ATAATAGTCCGCGCGCATCC |
| SSR64 | AACCCAGGAGAGCAAACCAT | ACCATCGTCGTCACTGTCAC |
|  | AACCCAGGAGAGCAAACCAT | CACCATCGTCGTCACTGTCA |
| SSR65 | GGATCTGATCATCGGATGGCT | GCTCCGAATCACCAAGACCT |
|  | GGATCTGATCATCGGATGGCT | AGCTCCGAATCACCAAGACC |
|  | AGGATCTGATCATCGGATGGC | GCTCCGAATCACCAAGACCT |
| SSR66 | GGCTGTGGCGAATTTGGAAA | CGGCGAACTGATCTTCTCCA |
|  | GGCTGTGGCGAATTTGGAAA | GCATCTCCCATGGGTTCCAT |
|  | GGCTGTGGCGAATTTGGAAA | CCCATGGGTTCCATCACAGT |
| SSR67 | CACGAGTTGTGTCCGGAATT | CGTTCTTTTCGTCGCTCTGC |
|  | CACGAGTTGTGTCCGGAATT | GCGTTCTTTTCGTCGCTCTG |
| SSR68 | GTTGGCTGGCATTCTTGTTCA | GGCATGCTGAAAAGCTCGTG |
|  | GTTGGCTGGCATTCTTGTTCA | GCATGCTGAAAAGCTCGTGA |
| SSR69 | TGCACATACAAAACACCTGACA | TGTGTGAGCTGCTCTTCTGC |
|  | CTGCACATACAAAACACCTGACA | TGTGTGAGCTGCTCTTCTGC |
|  | ACTGCACATACAAAACACCTGAC | TGTGTGAGCTGCTCTTCTGC |
| SSR70 | AACCAGCATGCTCAGATGTTG | AAGAATGAGACCAAGAAGTGTAGC |
|  | ATTAACCAGCATGCTCAGATGT | AATGAGACCAAGAAGTGTAGCCA |
|  | TTAACCAGCATGCTCAGATGT | GAATGAGACCAAGAAGTGTAGCC |
| SSR71 | CACACGTGTTTCTTGTCGCA | AGGATTGCAGCAGCTAGAGC |
|  | CACACGTGTTTCTTGTCGCA | CAGGAGGATTGCAGCAGCTA |
|  | CACACGTGTTTCTTGTCGCA | GGAGGATTGCAGCAGCTAGA |
| SSR72 | GATGTGGACGCCTTGTGTGT | CGCGGCTGAATACATACCCA |
|  | ATGTGGACGCCTTGTGTGT | CGCGGCTGAATACATACCCA |
|  | GATGTGGACGCCTTGTGTGT | TGCGCGGCTGAATACATACC |
| SSR73 | CACTGAAGTGCATTCATTCCTTGA | GTCATGAGGACAACGGACAA |
|  | CACTGAAGTGCATTCATTCCTTG | GTCATGAGGACAACGGACAA |
|  | CACTGAAGTGCATTCATTCCT | GTCATGAGGACAACGGACAA |
| SSR74 | CAGTGGGTTTGCTGCAACTT | TGGGCAGCCAGTCAATCAAA |
|  | TTCCAGTGGGTTTGCTGCAA | TGGGCAGCCAGTCAATCAAA |
|  | TTTCCAGTGGGTTTGCTGCA | TGGGCAGCCAGTCAATCAAA |
| SSR75 | ACAGTCGACCTCAAGCTCGA | GGTTATGGCTCGCCCTTTCA |
|  | TCGACCTCAAGCTCGACTTT | GGTTATGGCTCGCCCTTTCA |
|  | ACCTCAAGCTCGACTTTCTCG | GGTTATGGCTCGCCCTTTCA |
| SSR76 | CCGACGAGTTCCAGATCTGA | GAAGGTGGTGGTGGAGAAGG |
|  | TCCGACGAGTTCCAGATCTG | GAAGGTGGTGGTGGAGAAGG |
|  | CCGACGAGTTCCAGATCTGA | AGTGGTGGGCAAGATGAAGG |
| SSR77 | CGCTTTACGCATCCCAAAGG | GTGGAGAGGGAGAGCCAAAC |
|  | CGCTTTACGCATCCCAAAGG | TGGGAAGAGATCAGAGACGGT |
|  | CGCTTTACGCATCCCAAAGG | GATCAGAGACGGTGGAGAGG |
| SSR78 | GCACCTTCTTTGCTTGCGAT | CCTCTCTCGTGTTTGTGGCT |
|  | AAACGCAGCACCTTCTTTGC | CCTCTCTCGTGTTTGTGGCT |
|  | TCCAAAACCCCAAACCAGGA | CCTCTCTCGTGTTTGTGGCT |
| SSR79 | AACGCAGCTCCTCTCCAAAA | CTGCTGCTGTGTGTTGTAGC |
|  | AACGCAGCTCCTCTCCAAAA | GCTGCTGCTGTGTGTTGTAG |
|  | GCTCCTCTCCAAAACCCCAA | CTGCTGCTGTGTGTTGTAGC |
| SSR80 | TGAAAGAATCAGACATGGCT | AGCTGGTGTGGTAATCCTGT |
|  | TGAAAGAATCAGACATGGCT | TGAGCTGGTGTGGTAATCCT |
| SSR81 | GCAGCAAAAGGAGAGGGCTT | AGCTGCCTTGAAACCGTACA |
|  | AGCAAAAGGAGAGGGCTTCT | AGCTGCCTTGAAACCGTACA |
|  | CAGCAAAAGGAGAGGGCTTC | AGCTGCCTTGAAACCGTACA |
| SSR82 | TGGGTCACAGTGGTTGAAAA | TCAAGTCTGAGTTCCTTCAACACT |
|  | TGGGTCACAGTGGTTGAAAATG | TCAAGTCTGAGTTCCTTCAACACT |
|  | GGGTCACAGTGGTTGAAAATGT | TCAAGTCTGAGTTCCTTCAACACT |
| SSR83 | TCATCATCATCTGGTGTGTGCA | GCGAGTGGTAGTTAGGTAGGC |
|  | TGGTGTGTGCAAATCTGAATGC | GCGAGTGGTAGTTAGGTAGGC |
|  | GGTGTGTGCAAATCTGAATGC | GCGAGTGGTAGTTAGGTAGGC |
| SSR84 | ACCTCCAAGACTTGAACATCGT | AGGCAGCATTTCACCATTTCG |
|  | ACCTCCAAGACTTGAACATCGT | GGTGCGACTAAGTTGGAGAGT |
|  | TCCAAGACTTGAACATCGTCCT | AGGCAGCATTTCACCATTTCG |
| SSR85 | CTGCTGCAGCCATTAAAGGAG | GGCAGTTACAGGTCCTCCTC |
|  | TGCTGCAGCCATTAAAGGAG | GGCAGTTACAGGTCCTCCTC |
|  | CTGCTGCAGCCATTAAAGGA | GGCAGTTACAGGTCCTCCTC |
| SSR86 | TGCACCACGTACGCAATTTG | GCTAGCGCCCTCTTTCCTTT |
|  | TGCACCACGTACGCAATTTG | GACAGCTAGCGCCCTCTTTC |
|  | TGCACCACGTACGCAATTTG | TCGAGATGAGACAGCTAGCG |
| SSR89 | GTCAGAATGCCTCCTCTCTGA | AAAACAGGAGTGGGGACTGC |
|  | GTCAGAATGCCTCCTCTCTGA | GCAAAACAGGAGTGGGGACT |
|  | AGTCAGAATGCCTCCTCTCTG | AAAACAGGAGTGGGGACTGC |
| SSR90 | ACAGACGTTCGCGAGGTAAG | AATCCGCAACTACGCACGTA |
|  | CTACAGACGTTCGCGAGGTA | AATCCGCAACTACGCACGTA |
| SSR91 | TTTTCTTGCCGGTGGAGGTC | GGATCGGCAGCCATTAAACC |
|  | GTGGCAGAGAGGAGGTTCTT | GGATCGGCAGCCATTAAACC |
|  | TTTTCTTGCCGGTGGAGGTC | AGAATAAGGATCGGCAGCCA |
| SSR92 | GCAACCACCAACAGATGACG | GCTTCTTGTTTCCAGGCAAGA |
|  | ACCAGCGGCAACATCCTTAT | GCTTCTTGTTTCCAGGCAAGA |
| SSR93 | TCAACAGCAGGCGTCTTCTT | TGCCGAAGCAGCATCCATTA |
|  | ATGGCAGCAGCAGATGAGAG | TGCCGAAGCAGCATCCATTA |
| SSR94 | CTTGATCCTTCTGCTCCGTCT | CCTTCTTGTGGTGCTAACTGC |
|  | TTGATCCTTCTGCTCCGTCT | CCTTCTTGTGGTGCTAACTGC |
| SSR95 | TATCGTCAACCACCGGCTTC | GCGTCTGTTTAGGCATTGAAGG |
|  | CGGCTTCACAAACGTTCTCG | GCGTCTGTTTAGGCATTGAAGG |
|  | TATCGTCAACCACCGGCTTC | TGCGTCTGTTTAGGCATTGA |
| SSR96 | GGTTACAGCGGCAACAATGG | TGTTGCTGCTGTTAGTCCGT |
|  | GCAACAATGGTGGAGGTCCT | TGTTGCTGCTGTTAGTCCGT |
|  | GGAGGTCCTTCACGGTAGGA | TGTTGCTGCTGTTAGTCCGT |
